# Preoperative multimodal CT for selection of acute anterior circulation occlusion stroke patients for mechanical thrombectomy

**DOI:** 10.3389/fneur.2026.1818377

**Published:** 2026-05-08

**Authors:** Guiyun Luo, Manhong Deng, Lilan She, Guiling Chen

**Affiliations:** 1 Department of Neurology, Sanming First Hospital Affiliated to Fujian Medical University, Sanming, China; 2 Department of Radiology, Sanming First Hospital Affiliated to Fujian Medical University, Sanming, China; 3 Department of Radiology, Fujian Medical University Union Hospital, Fuzhou, China

**Keywords:** acute ischemic stroke, collateral score, large vessel occlusion, mechanical thrombectomy, multimodal CT

## Abstract

**Objective:**

By developing and comparing various preoperative multimodal CT-based models to predict prognosis after successful recanalization in acute anterior circulation occlusive stroke, this study aimed to ascertain the prognostic assessment of patients following successful recanalization.

**Methods:**

Patients with acute anterior circulation large vessel occlusion who underwent mechanical thrombectomy and achieved successful recanalization were consecutively enrolled from Sanming First Hospital Affiliated to Fujian Medical University between January 2022 and October 2024. Based on the 90-day modified Rankin Scale (mRS) scores, the patients were categorized into a favorable clinical outcome group (mRS 0–3) and an unfavorable clinical outcome group (mRS 4–6). Following the identification of statistically significant variables from the analyzed clinical and imaging data, optimal cut-off values were computed. Five multivariate logistic regression models were subsequently constructed: Clinical-Imaging (C-I), Clinical-non-Perfusion (C-NP), Clinical-non-Angiography (C-NC), Clinical-only (C), and Imaging-only (I). Models performance were compared using receiver operating characteristic (ROC) curve analysis, with comparisons based on the area under the curve (AUC), sensitivity, and specificity. Using the Delong test for model comparison and selection, a nomogram of the optimal model was developed. Model performance was subsequently assessed by means of a confusion matrix and 5-fold cross-validation.

**Results:**

Of the 131 enrolled patients, 77 (58.78%) were classified into the favorable clinical outcome group and 54 (41.22%) into the unfavorable clinical outcome group. Statistically significant differences were identified in age (59.5 years), preoperative blood glucose (PBG) (7.21 mmol/L), National institutes of health stroke scale (NIHSS) (18.5), Alberta Stroke Program Early CT Score (ASPECTS) (7.5), collateral score (3.5), infarct core volume (19.25 mL), and hypoperfusion volume (180.65 mL). The AUCs for the five models were C-I Model (0.851, 95% CI: 0.785–0.917), C-NC Model (0.865, 95% CI: 0.804–0.926), C-NP Model (0.861, 95% CI: 0.798–0.923), C Model (0.713, 95% CI: 0.626–0.801) and I Model (0.772, 95% CI: 0.688–0.855). The accuracy of the optimal C-NP Model was 0.771, and the mean AUC from 5-fold cross-validation was 0.828.

**Conclusion:**

The combined model revealed strong predictive power regarding outcomes following successful mechanical thrombectomy. Furthermore, the C-NP Model holds greater practical utility, as it enables clinicians to quickly assess the prognosis after a successful intervention.

## Introduction

Stroke is characterized by high incidence, mortality, and disability rates, imposing a substantial economic burden, and represents the leading cause of death and disability among Chinese adults (1, 2). Acute ischemic stroke (AIS), the most common type, is attributable to large vessel occlusion in 35–40% of cases, which is responsible for 95.6% of all stroke-related deaths (3, 4). Mechanical thrombectomy (MT) has been established as the most effective treatment for large vessel occlusive stroke (LVOS) (5–11). However, despite achieving high recanalization rates of 71–95%, approximately 45–54% of patients experience unfavorable clinical outcome, resulting in poor clinical outcomes (12–16). Unfavorable clinical outcome can be attributed to a variety of factors, such as patient age, preoperative blood glucose (BPG), National institutes of health stroke scale (NIHSS) score, preoperative imaging characteristics, the number of thrombectomy passes, and postoperative management strategies (17, 18). This time sensitivity is underscored by evidence showing that a mere one-hour delay in achieving recanalization reduces the chance of a good clinical prognosis by approximately 38% (19). Hence, it is crucial to rapidly provide a preliminary assessment of the likelihood of a favorable postoperative outcome based on limited preoperative data. This assessment is instrumental in facilitating rapid consensus between clinicians and patients’ families regarding the surgical decision.

Although both multimodal CT and MRI are used for pre-MT assessment (20, 21), CT is often more practical and widely accessible than MRI in the acute stroke setting, given the latter’s relative contraindications and limited availability in many institutions. Multimodal CT primarily includes non-contrast CT (NCCT), CT angiography (CTA), and CT perfusion (CTP). NCCT is primarily used to assess for the presence of intracranial hemorrhage, as well as the location and extent of infarction. CTA is employed to determine the site of vascular occlusion and evaluate collateral compensation. CTP provides information on the volume of the infarct core and the ischemic penumbra. The acquisition of complete multimodal CT, especially CTP, is often challenging in severe stroke due to poor patient cooperation. Moreover, performing CTP on AIS patients without large vessel occlusion, who will not undergo MT, results in unnecessary radiation exposure. To address this, our study aims to develop preoperative models combining different clinical and imaging variables. We seek to explore their predictive ability for the prognosis of successful mechanical thrombectomy, ultimately providing a basis for clinicians and patients to decide whether to undergo intervention.

## Materials and methods

### Study strategy and data collection

A consecutive series of patients with acute anterior circulation occlusion who underwent MT and achieved successful recanalization at Sanming First Hospital Affiliated to Fujian Medical University between January 2022 and October 2024 were retrospectively enrolled. All patients underwent preoperative multimodal CT imaging. Prior to the examination, the purpose and procedures of the study were explained to the patients and/or their families, and written informed consent was obtained from all participants. This study was reviewed and approved by the Ethics Committee of Sanming First Hospital Affiliated to Fujian Medical University.

**Figure 1:**
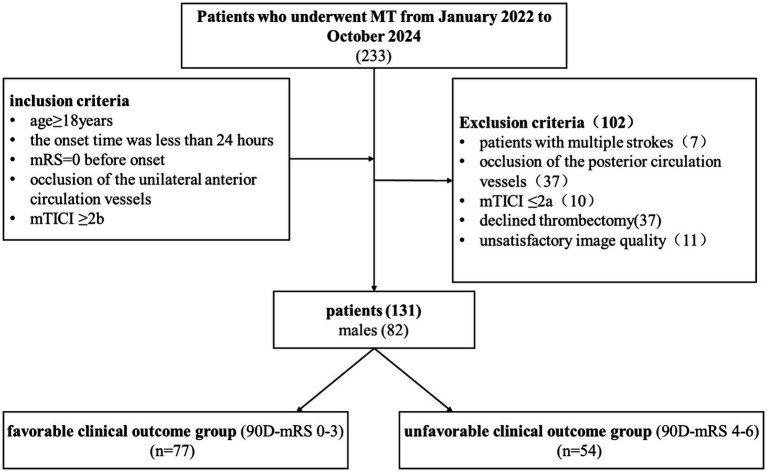
Flowchart of the enrolled patients in our study. mRS, Modified Rankin score; mTICI, modified thrombolysis in cerebral infarction; 90D-mRS, 90-day modified Rankin score from onset.

The inclusion criteria were as follows: (1) age ≥18 years; (2) time from onset to treatment ≤ 24 h; (3) a pre-stroke modified Rankin Scale (mRS) score of 0 (22); (4) CTA-confirmed unilateral acute anterior circulation large vessel occlusion, including the internal carotid artery (ICA), the A1 segment of the anterior cerebral artery (ACA), and the M1-M3 segments of the middle cerebral artery (MCA); (5) successful post-procedural recanalization (mTICI ≥ 2b) (23, 24). Patients were excluded for any of the following reasons: (1) a history of multiple strokes; (2) presence of posterior circulation occlusion; (3) unsuccessful recanalization (mTICI ≤ 2a); (4) patient refusal or lack of recommendation for thrombectomy; (5) poor image quality (Figure 1).

### Research method

#### CT protocol

Imaging was acquired on the Revolution CT system (GE Healthcare, United States) utilizing a low-dose protocol with adaptive statistical iterative reconstruction (level = 50%). The imaging protocol was as follows and was completed in one continuous session lasting approximately 10 min: head NCCT, followed by head-and-neck CTA, multiphase CTA (mCTA) of the intracranial vasculature, and finally, whole-brain CTP. The detailed scan parameters are summarized in Table 1.

**Table 1:** Multimodal CT scan protocols.

Parameter	NCCT	Head and neck CTA/mCTA	CTP
Scan mode	Axial	Helical	Axial (Cine)
Detector coverage (mm)	160	–	160
Rotation time (s)	1	0.5	0.5
Tube voltage (kV)	120	100	80
Tube current (mA)	Automated (200–300)	Automated (80–350)	Fixed (130)
Pitch	–	0.992:1	–
Slice thickness (mm)	1.25	1.25	5.0
Contrast volume (mL)	–	50	40
Saline flush (mL)	–	30	30
Injection rate (mL/s)	–	5	5
Acquisition timing	–	Arterial phase from aortic arch to vertex; mCTA: 2 additional scans at 8 s intervals at skull base-vertex.	12 scans @ 2 s interval, then 8 scans @ 3 s interval, post 5–6 min delay.

mCTA, multiphase CTA.

#### Imaging post-processing

The NCCT, CTA, and mCTA images were post-processed on an Advantage Workstation 4.7 (GE Healthcare) utilizing the *FastStroke* application. This software was used to create axial maximum intensity projection (MIP) images with a 20-mm slab thickness, yielding separate arterial, venous, and late venous phase images of the cerebral vasculature and their corresponding color-fused MIP maps, where the three phases were assigned red, green, and blue, respectively.

The raw CTP images were analyzed by the Computer-Aided Analysis System for Cerebral CTP Imaging (Shanghai United Imaging Intelligence Co., Ltd.). The system automatically generated perfusion parameter maps. If the time-density curve was aberrant, the arterial input (M1 segment of the unaffected middle cerebral artery) and venous outflow (Torcular Herophili) were manually redefined before the data were reprocessed.

#### Data collection

Patients’ clinical data were recorded, including sex, age, and history of hypertension, diabetes mellitus, coronary heart disease, and atrial fibrillation. PBG levels and the NIHSS scores were also documented. The Alberta stroke program early CT score (ASPECTS) was determined from the head NCCT images. The length of the occluded vessel and the collateral score were obtained from the head-and-neck CTA and mCTA datasets. Based on CTP, the following volumetric parameters were derived: hypoperfusion volume (Tmax > 6 s), infarct core volume (CBF < 30%), and the ischemic penumbra volume (defined as the mismatch between the two).

Two radiologists with more than a decade of experience independently performed all quantitative and qualitative image analyses under blinded conditions, with interobserver consistency assessed by the intraclass correlation coefficient (ICC). The mean values of continuous measurements from both readers were adopted for further statistical analysis. In case of disagreements on ordinal ratings, a final consensus was reached through deliberation.

### Follow-up and outcomes

The 90-day modified Rankin Scale (90D-mRS) score was assessed by a neurologist through either outpatient visits or telephone interviews. Based on the 90D-mRS scores, patients were stratified into two groups: a favorable clinical outcome group (scores 0–3) and an unfavorable clinical outcome group (scores 4–6).

### Statistical analysis

We performed statistical analyses with R software (v4.5.1) and IBM SPSS Statistics 25, setting the significance level at *p* < 0.05. For univariate comparisons, categorical variables were analyzed using Pearson’s chi-square or Fisher’s exact test. Continuous variables were first tested for normality with the Shapiro–Wilk test; those normally distributed were compared with an independent *t*-test, and non-parametric data were compared with the Mann–Whitney *U* test. Optimal cut-off values were determined for the statistically significant variables. These variables were then included in a multivariable logistic regression analysis to construct predictive models. The predictive performance of the models was compared using receiver operating characteristic (ROC) curve analysis by examining the area under the curve (AUC), sensitivity, and specificity. Using the Delong test for model comparison and selection, a nomogram of the optimal model was developed. Model performance was subsequently assessed by means of a confusion matrix and 5-fold cross-validation.

## Results

### Baseline data analysis

Among the 131 enrolled patients, 77 (58.78%) were classified into the favorable clinical outcome group and 54 (41.22%) into the unfavorable clinical outcome group. Among them, severe postoperative intracranial hemorrhage occurred in 5 patients, 3 of whom died. Overall, 14 patients died (90D-mRS 6), accounting for 10.7% of the total and 25.9% of the unfavorable clinical outcome group. The ICC values for length of thrombus, ASPECTS, and collateral score were 0.998 (95%CI: 0.997–0.998), 0.907 (95%CI: 0.871–0.933), 0.877 (95%CI: 0.830–0.911), respectively. These ICC values demonstrate substantial interobserver agreement for all three imaging variables, supporting the robustness of our image-based predictors. Compared with patients in the favorable clinical outcome group, those in the unfavorable clinical outcome group were older (*p* = 0.001), had higher PBG and NIHSS scores (*p* = 0.028 and *p* = 0.007, respectively), lower ASPECTS and collateral scores (both *p* < 0.001), and larger infarct core volumes and hypoperfusion volumes (*p* < 0.001 and *p* = 0.029, respectively). The optimal cutoff values for these parameters were 59.5 years (age), 7.21 mmol/L (PBG), 18.5 (NIHSS), 7.5 (ASPECTS), 3.5 (collateral score), 19.25 mL (infarct core volume), and 180.65 mL (hypoperfusion volume), as presented in Tables 2, 3.

**Table 2:** Univariate analysis of all data.

Variate	Favorable (*n* = 77) (%)	Unfavorable (*n* = 54) (%)	*X^2^*/*t*/*Z*	*P-*value
Male	46(59.7)	36(66.7)	0.650	0.420
Age (y)	64.3 ± 10.8	70.7 ± 9.7	0.416	0.001
Hypertension	43(55.8)	32(59.3)	0.151	0.697
Diabetes	14(18.2)	16(29.6)	2.356	0.125
AF	26(33.8)	22(40.7)	0.665	0.415
PBG (mmol/L)	6.35(5.72, 7.27)	7.24(5.94, 9.87)	−2.190	0.028
NIHSS	15 ± 6	18 ± 6	1.628	0.007
Length of thrombus (mm)	17(12, 28)	19.5(13, 29.5)	−1.383	0.167
ASPECTS	8(7, 9)	6(5, 8)	−4.725	<0.001
Collateral score	4(3, 4)	3(2, 3)	−4.916	<0.001
Infarct core volume (ml)	3.9(0.7, 15.0)	26.3(8.9, 75.8)	−5.286	<0.001
Hypoperfusion volume (ml)	138.4(92.0, 174.1)	156.6(93.4, 245.2)	−2.179	0.029
Ischemic penumbra volume (ml)	119.8(83.0, 159.4)	108.8(73.7, 141.8)	−0.650	0.516

AF, atrial fibrillation; PBG, preoperative blood glucose; NIHSS, national institutes of health stroke scale; ASPECTS, Alberta stroke program early CT score; *X*^2^/*t*/*Z*, statistical value.

**Table 3:** The optimal cut-off value of the effective variable.

Variate	Sensitivity	Specificity	Maximum Youden index	OCV
Age	0.870	0.390	0.260	59.5
PBG	0.519	0.753	0.272	7.21
NIHSS	0.593	0.688	0.281	18.5
ASPECTS	0.688	0.722	0.411	7.5
Collateral score	0.597	0.815	0.412	3.5
Infarct core volume	0.648	0.818	0.466	19.25
Hypoperfusion volume	0.463	0.818	0.281	180.65

PBG, preoperative blood glucose; NIHSS, national institutes of health stroke scale; ASPECTS, Alberta stroke program early CT score; OCV, optimal cut-off values.

### Model building

**Figure 2:**
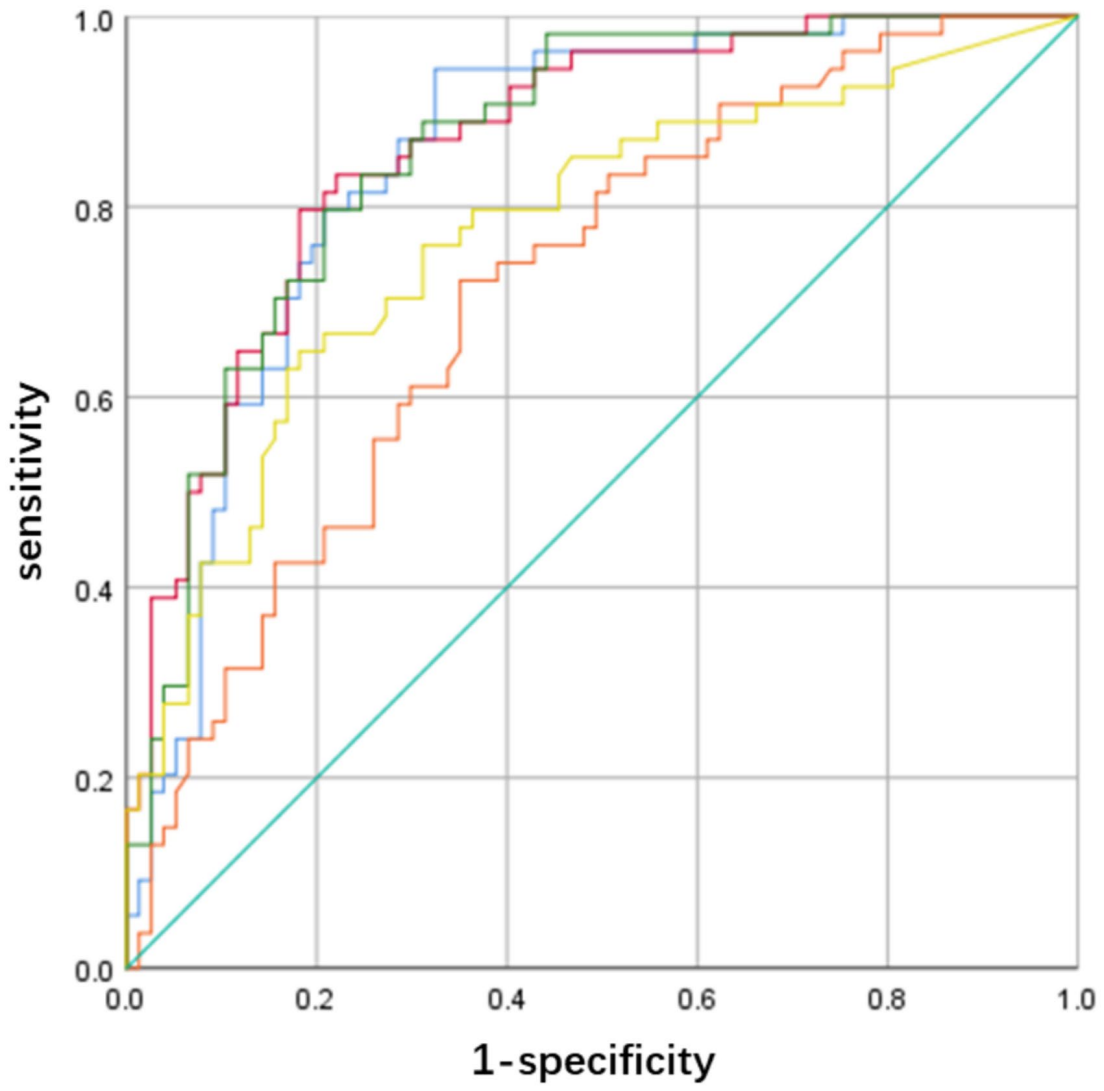
Receiver operating characteristic (ROC) curves of the five models. Blue for C-I Model; Red for C-NC Model; Green for C-NP Model; Orange for C Model; Yellow for I Model.

Variables with *p* < 0.05 in the baseline data analysis were included in a multivariable logistic regression analysis to construct five distinct models: a clinical-imaging model (C-I Model), a clinical-non-collateral score model (C-NC Model), a clinical-non-perfusion model (C-NP Model), a comprehensive clinical model (C Model), and a comprehensive imaging model (I Model), as detailed in Table 4. Receiver operating characteristic (ROC) curves were plotted for each model. The area under the curve (AUC), sensitivity, and specificity were calculated based on these ROC curves, with the results presented in Table 5 and Figure 2.

**Table 4:** Multivariate logistic regression analysis of five models.

Variate	C-I model			C-NC model			C-NP model		
	*β*	OR (95%CI)	*P*	*β*	OR (95%CI)	*P*	*β*	OR (95%CI)	*p* value
Age	0.108	1.114 (1.053, 1.177)	<0.001	0.110	1.116 (1.055, 1.181)	<0.001	0.109	1.116 (1.057, 1.178)	<0.001
PBG (mmol/L)	0.119	1.127 (1.001, 1.268)	0.048	0.124	1.131 (1.006, 1.273)	0.039	0.120	1.128 (1.003, 1.268)	0.044
NIHSS	0.024	1.026 (0.952, 1.105)	0.505	0.025	1.026 (0.952, 1.104)	0.506	0.030	1.030 (0.956, 1.110)	0.432
ASPECTS	−0.436	0.646 (0.465, 0.898)	0.009	−0.502	0.606 (0.443, 0.828)	0.002	−0.504	0.604 (0.441, 0.826)	0.002
Collateral score	−0.403	0.668 (0.313, 1.426)	0.298	–	–	–	−0.696	0.499 (0.251,0.989)	0.047
Infarct core volume	0.016	1.016 (0.996, 1.036)	0.119	0.019	1.019 (1.001, 1.038)	0.043	–	–	–
Hypoperfusion volume	−0.001	0.999 (0.993, 1.005)	0.755	−0.001	0.999 (0.993, 1.005)	0.726	–	–	–

Variate	C model			I model		
	*β*	OR (95%CI)	*P*	*β*	OR (95%CI)	*P*
Age	0.065	1.068 (1.026, 1.111)	0.001	–	–	–
PBG (mmol/L)	0.100	1.105 (0.991, 1.231)	0.071	–	–	–
NIHSS	0.073	1.076 (1.009, 1.148)	0.025	–	–	–
ASPECTS	–	–	–	−0.234	0.792 (0.608, 1.031)	0.083
Collateral score	–	–	–	−0.542	0.581 (0.298,1.134)	0.111
Infarct core volume	–	–	–	0.021	1.021 (1.002, 1.041)	0.030
Hypoperfusion volume	–	–	–	−0.001	0.999 (0.993, 1.005)	0.635

PBG, preoperative blood glucose; NIHSS, national institutes of health stroke scale; ASPECTS, Alberta stroke program early CT score.

**Table 5:** Prediction efficiency of the five models.

Model	AUC	95%CI	Sensitivity	Specificity
C-I	0.851	0.785–0.917	0.944	0.675
C-NC	0.865	0.804–0.926	0.796	0.818
C-NP	0.861	0.798–0.923	0.796	0.792
C	0.713	0.626–0.801	0.722	0.649
I	0.772	0.688–0.855	0.648	0.818

AUC, area under curve.

#### Model evaluation

**Figure 3:**
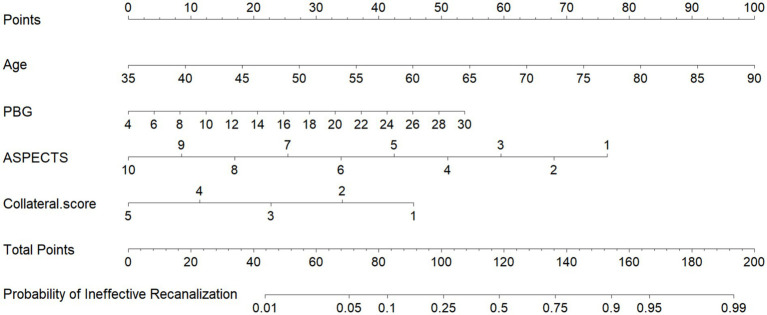
Nomogram of the C-NP model.

**Figure 4:**
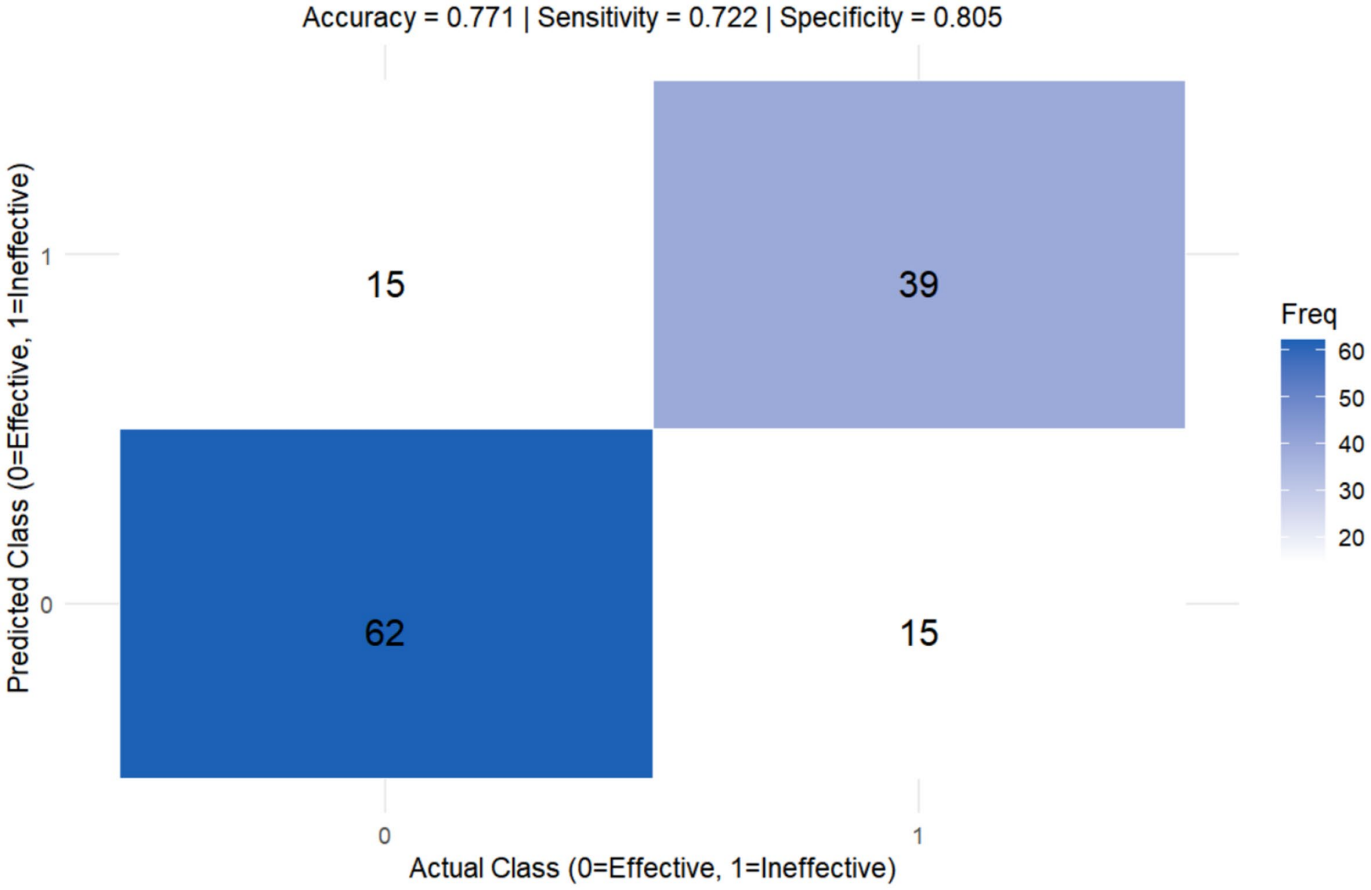
Confusion matrix of C-NP model.

**Figure 5:**
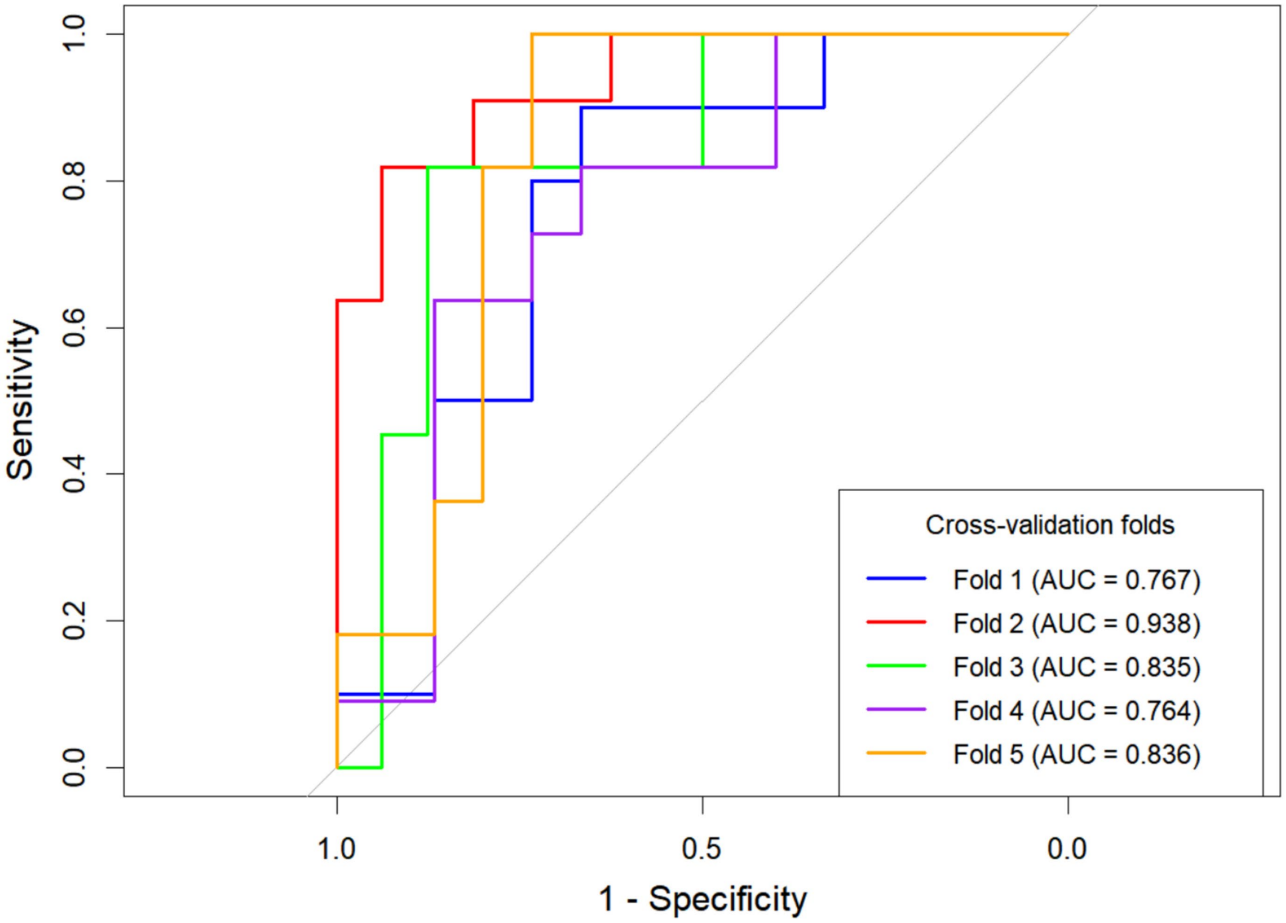
5-fold cross-validation of C-NP model.

**Figure 6:**
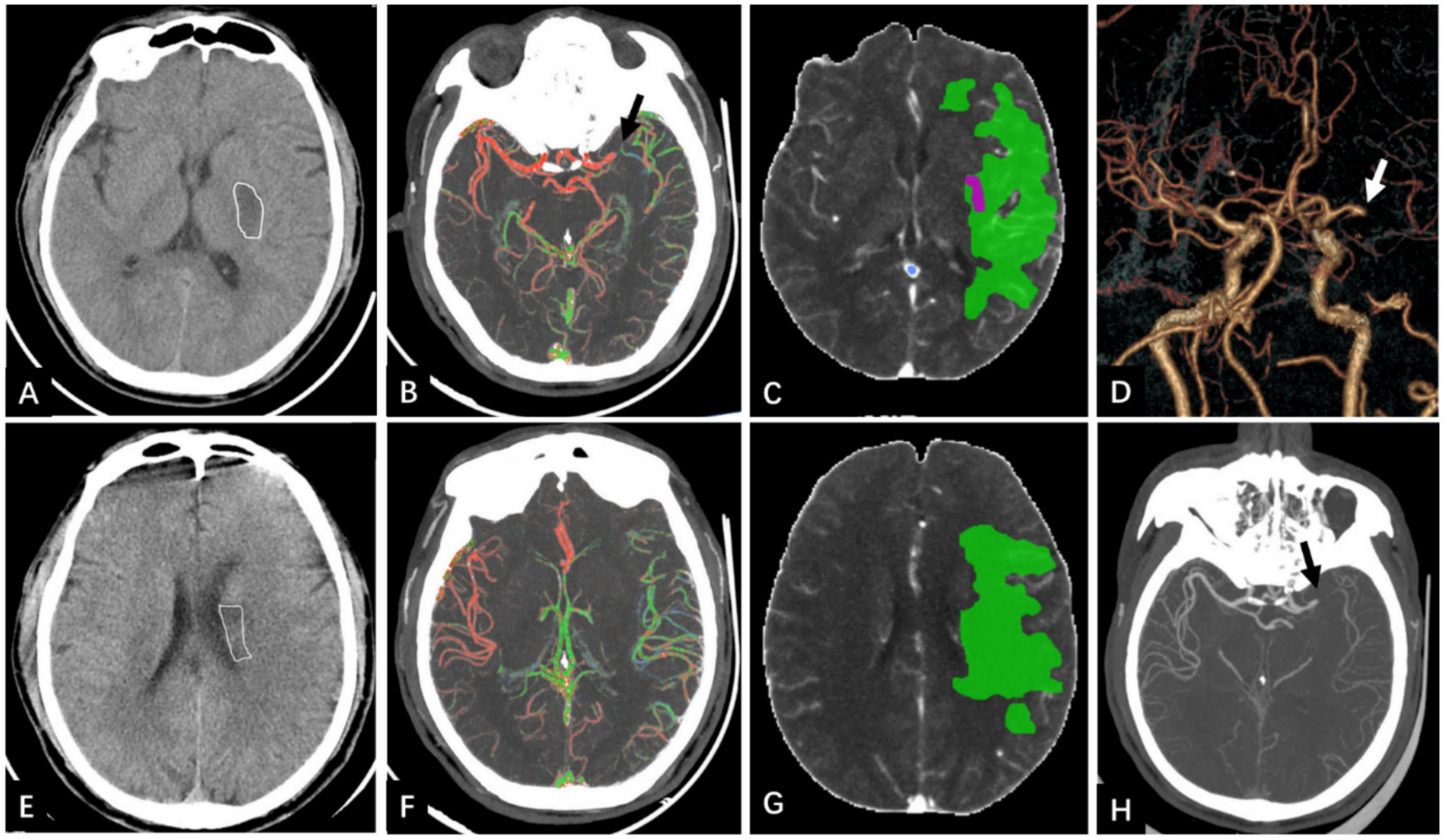
A 55-year-old man presented with “right limb weakness for more than 8 hours”. BPG = 6.21 mmol/L, reclassified as the favorable clinical outcome group with 90-mRS score 1 point. **(A,E)** NCCT, showing slightly reduced density of the left lenticular nucleus (map **A**) and caudate nucleus (map **E**), ASPECTS is 8 points. **(B,F)** Color-coded mCTA, show that the number of blood vessels in the left MCA supply area is not reduced, and green blood vessels can be seen, blood vessel display is delayed by 1 phase, and the collateral score is 4 points. **(C,G)** The CTP maps generated by Computer-Aided Analysis System for Cerebral CTP Imaging. The red part is the infarct core area, and the volume is 1.2 mL. The green part is the IP area, and the volume is 90.7 mL. The hypoperfusion volume is 91.9 mL of the sum of the two. **(D,H)** The head CTA, showing that the occluded vessel was the M1 segment of the left MCA (white and black arrows), and the length of thrombus was 11 mm. According to the nomogram, a total score of 67.5 points—comprising Age (36.25 points), BPG (3) ASPECTS (17 points) and collateral score (11.25 points)—corresponds to a predicted risk less than 10%.

**Figure 7:**
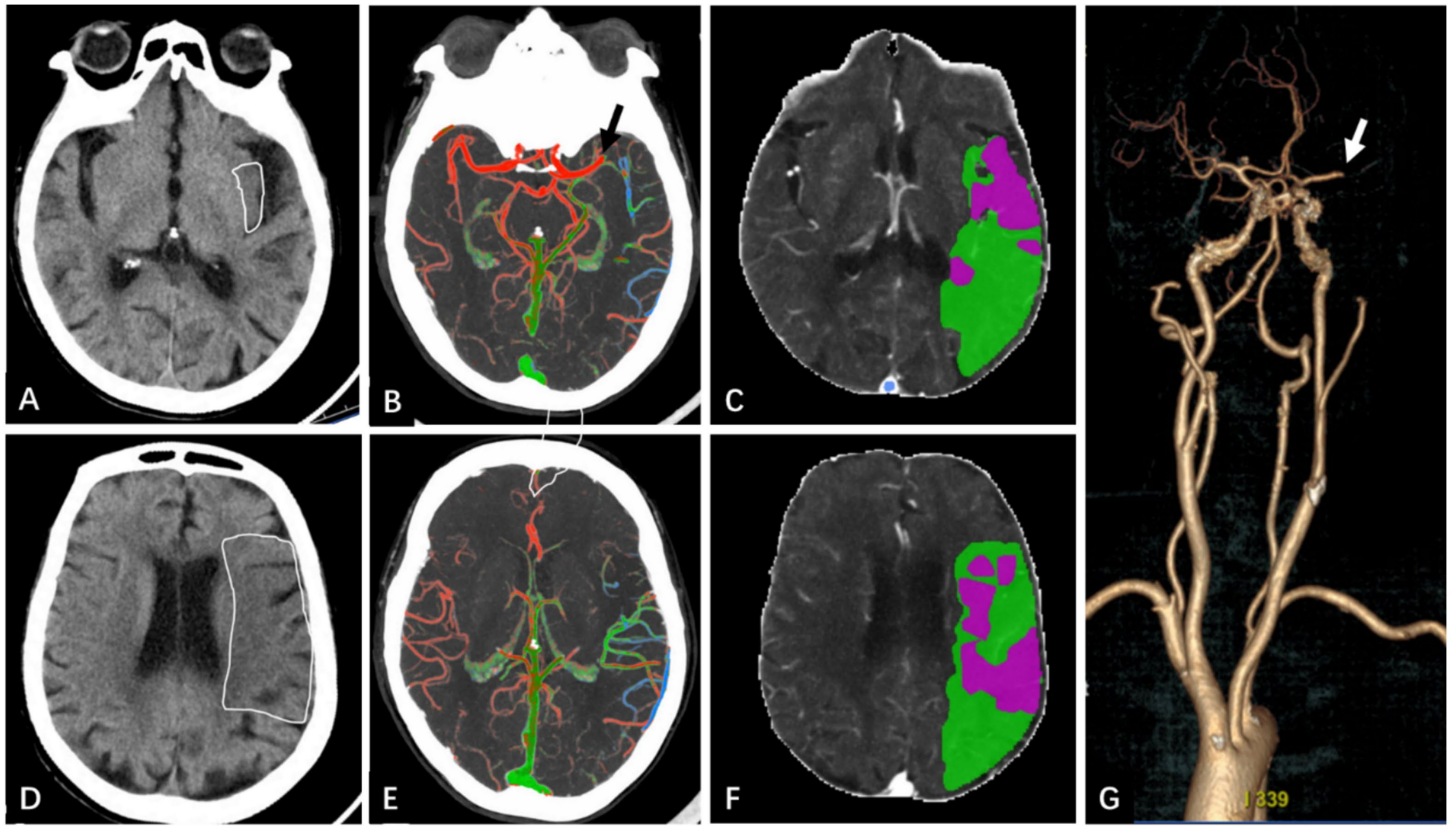
A 86-year-old man presented with “right-sided limb weakness and aphasia for over 3 h.”. BPG = 12.1 mmol/L, reclassified as the unfavorable clinical outcome group with 90-mRS score 5 point. **(A,D)** NCCT, the underlined areas show reduced density in the left insula (map **A**) and the anterior, lateral, and posterior MCA territories immediately superior to basal ganglia (map **D**), ASPECTS is 6 points. **(B,E)** Color-coded mCTA, showing green and blue blood vessels in the left MCA blood supply area, blood vessel was display delayed by 2 phases, and the collateral score is 3 points. **(C,F)** The CTP maps generated by Computer-Aided Analysis System for Cerebral CTP Imaging. The red part is the infarct core area, and the volume is 30.6 mL. The green part is the IP area, and volume is 105.1 mL. The low perfusion volume is 135.7 mL. Panel **(G)** shows the head and neck CTA. The occluded vessels was the M1 segment of the left MCA (white arrows), and the length of thrombus was 7 mm. According to the nomogram, a total score of 167 points—comprising Age (92.5 points), BPG (17.5) ASPECTS (34 points) and collateral score (23 points)—corresponds to a predicted risk about 95%.

A comparison of the AUC values among the five models reveals that the combined models outperform the individual models. The C-NC Model and the C-NP Model achieved the highest AUCs and were therefore selected for further comparison. Delong’s test showed no statistically significant difference in the predictive performance between the C-NC Models and C-NP Models (*Z* = 0.334, *p* = 0.739). In clinical practice, however, the C-NP model streamlines the workflow and provides essential cerebrovascular information, making it more suitable for interventional intervention assessment. Based on the above findings, we constructed a nomogram for the C-NP model and presented its corresponding confusion matrix (Figures 3, 4). The accuracy of the optimal C-NP Model was 0.771, and the sensitivity and specificity were 0.722 and 0.805, respectively. We employed 5-fold cross-validation to reassess the performance of our model (Figure 5). This process yielded a set of five performance metrics, from which the mean and standard deviation were calculated. The mean AUC from 5-fold cross-validation was 0.828. Given the limited number of outcome events (*n* = 54), our models face a potential risk of overfitting. The moderate variability in cross-validated AUCs (SD 0.065) suggests some model instability. Therefore, our findings should be considered hypothesis-generating, and external validation in larger independent cohorts is necessary before clinical application. We demonstrate the application of our nomogram for personalized risk prediction with case examples (Figures 6, 7).

## Discussion

Endovascular therapy has made remarkable progress, with continual innovation in devices leading to a significant increase in recanalization rates for LVOS patients. However, favorable outcomes remain elusive for about half of all treated patients, and a substantial number suffer from neurological deterioration or mortality (12, 25). By retrospectively collecting preoperative patient data, we developed and compared five models to evaluate their predictive value for prognosis after successful recanalization following MT, aiming to provide evidence for initial decision-making during preoperative discussions. Our results demonstrated that the combined model offered the best performance in predicting post-MT prognosis, thereby helping to avoid unnecessary surgical interventions.

In our study, the incidence of poor outcomes after MT in patients with anterior circulation LVOS was 41.22%, which is consistent with previous reports. Among them, the death toll accounted for 10.7% of the total and 25.9% of the unfavorable clinical outcome group. The predictors of death may differ from those of severe disability (90D-mRS 4–5). For example, while large hypoperfusion volume and older age might predict both outcomes, other factors such as post-recanalization hemorrhage, malignant cerebral edema, or systemic complications could be more specific for mortality (17, 18). Future research should more carefully distinguish between mortality and disability to better elucidate prognostic factors and guide clinical decision-making.

While most studies consider a 90D-mRS score of 0–2 as indicative of a favorable outcome in patients with acute ischemic stroke (AIS), some research has shown that AIS patients with 90D-mRS scores of 2 and 3 exhibit similar 7-year survival rates (26). Additionally, data suggest that AIS patients with 90D-mRS scores of 2 and 3 have comparable health-related quality of life scores (27). Therefore, in our study, categorizing a 90D-mRS score of 0–3 as representing a favorable outcome appears more appropriate for patients with large vessel occlusion. This is beneficial for preoperative risk–benefit assessments with patients and families who may consider moderate disability an acceptable outcome.

In the combined model of this study, age and PBG were identified as independent risk factors for futile recanalization following MT. Within the HIAT-2 score, an age greater than 59 years is associated with an increased risk of unfavorable outcomes (28). A study focusing on acute LVOS in the Chinese population indicated that an age younger than 66 years is associated with favorable prognosis (16). In our study, the optimal cutoff value for age was determined to be 59.5 years, which aligns with the aforementioned findings. Hyperglycemia increases lactate production by cerebral cells in the ischemic penumbra, leading to acidosis that exacerbates brain injury and expands the infarct volume. Furthermore, it impairs cerebral autoregulation, predisposing patients to reperfusion injury, and increases insulin resistance, thereby intensifying inflammatory and oxidative stress responses. Consequently, hyperglycemia serves as an independent risk factor for poor prognosis after MT (29, 30). Multiple studies on outcomes following successful thrombectomy for anterior circulation strokes have consistently shown that elevated PBG is associated with unfavorable outcomes (31, 32). Goyal’s study demonstrated that admission hyperglycemia was associated with a higher likelihood of 3-month mortality and lower odds of 3-month functional improvement in terms of shift in mRS score in ELVO patients treated with MT (33). The study by Filipov demonstrated that hyperglycemia on admission, rather than a history of diabetes, is an independent predictor of poor prognosis after MT in patients with anterior circulation large vessel occlusion (34). Our findings are consistent with this result. Therefore, patients with advanced age and elevated PBG require closer attention and stricter intraoperative as well as postoperative management.

The NIHSS score reflects the severity of stroke symptoms and serves as an important indicator in various prognostic prediction models (25, 28, 35). Patients who develop malignant middle cerebral artery infarction after intervention tend to have higher preoperative NIHSS scores (36). In the univariate analysis of this study, the NIHSS score was a significant assessment indicator and remained an important predictor in C Model. However, it showed no statistically significant difference in the combined models. We speculate that it may correlate with imaging indicators, which warrants further investigation with larger sample sizes.

The ASPECTS is a semi-quantitative method for assessing the volume of cerebral infarction. Brain tissue appearing as hypodense areas on NCCT is considered to represent irreversible ischemic injury (37, 38). Research by Haussen et al. (39) demonstrated that the NCCT-based ASPECTS is inversely correlated with the infarct core volume on CTP. Furthermore, studies have shown that an ASPECTS ≥7 corresponds to an infarct core volume of <70 mL, while an ASPECTS <4 corresponds to a volume >100 mL, indicating that ASPECTS can quantitatively reflect the size of infarct core (40). Raza et al. (41) replaced the NCCT-based ASPECTS in the PRE model—which originally comprised age, NIHSS, and NCCT-based ASPECTS—with infarct core volume and reevaluated the prognosis of AIS patients. They found that this substitution did not improve prognostic performance, indicating that ASPECTS and infarct core volume have comparable prognostic value for patient outcomes (41). In our study, NCCT-based ASPECTS showed statistical significance in combined models, with an optimal cutoff value of 7.5, suggesting that an ASPECTS ≥8 points is associated with favorable prognosis. These findings are generally consistent with the aforementioned research.

Multiple studies have indicated that infarct core volume is more dependent on collateral status, with accelerated progression of cerebral infarction occurring under poor collateral conditions (42–44). Therefore, collateral status serves as a critical factor for revascularization in patients beyond the standard time window. In recent years, collateral status assessment methods based on CTA or DSA have been widely used in patients with AIS. Among these, the Menon scoring system based on mCTA has been extensively studied and validated (45, 46). Studies have shown that the Menon score outperforms single-phase CTA and is comparable to simplified CTP in predicting clinical outcomes, while avoiding complex post-processing (47). Therefore, this study adopted the Menon scoring method to evaluate the collateral status of patients. Research has indicated that poor collateral status is one of the reasons why patients with LVOS do not undergo MT and is the baseline imaging feature most strongly associated with poor outcomes in wake-up stroke patients (48). In our study, the C-NP Model demonstrated that collateral score is an independent risk factor for predicting ineffective recanalization after MT, which is consistent with previous findings. It exhibits predictive capability comparable to the C-NC Model and is more suitable for patients unable to cooperate with imaging examinations.

Raza and Rangaraju (49) further demonstrated that infarct core volume, rather than ischemic penumbra volume, serves as a significant predictor of outcome in patients with LVOS. Infarct core volume is a key criterion for selecting patients for MT. For instance, the SWIFT PRIME trial excluded patients with an infarct core volume exceeding 50 mL; the DAWN trial required an infarct core volume no greater than 51 mL; and the DEFUSE 3 trial stipulated an infarct core volume below 70 mL (8, 10, 11). These criteria underscore that infarct core volume is the most critical variable among perfusion parameters. In the present study, infarct core volume emerged as an important parameter in the C-NC Model and was the sole variable in the I Model, indicating its essential role in predicting post-MT prognosis. Furthermore, the C-NC model achieved the highest AUC, highlighting the pivotal significance of infarct core volume in imaging assessment. These findings are consistent with previous research.

In a recent multinational survey, factors most strongly associated with the decision to perform MT included stroke severity (NIHSS score), patient age, guideline-based evidence level, pre-procedural brain imaging, and physician experience (50). Multiple studies have demonstrated that combined models integrating clinical and imaging variables can effectively predict post-MT outcomes (51–54). Consistent with previous findings, the combined models in the present study demonstrated the highest predictive performance for post-procedural prognosis. The HIAT-2 score, a classic predictor of poor outcome following anterior circulation intra-arterial therapy, incorporates age, pre-treatment glucose level, NIHSS score, and ASPECTS, achieving an AUC of 0.748 for predicting poor outcome (28). Another model based solely on imaging variables—comprising the clot burden score, multisegment clot burden, CBV-ASPECTS, and collateral score—yielded an AUC of 0.714 for predicting unfavorable outcomes post-MT (55). This indicates that combined models generally outperform models based solely on imaging data. Similarly, in the current study, the AUC of the combined models were also higher than those of the purely clinical or imaging-based models, which aligns with the findings from the aforementioned research.

Recent large-scale trials focusing on large infarct cores have increasingly favored simplified imaging protocols to enable rapid assessment (56, 57). Although MT provides clinical benefits for critically ill patients with large cerebral infarctions, it does not significantly reduce mortality (58). Our C-I model, which incorporates only NCCT examination, may therefore be better suited for the rapid evaluation of patients with large infarct cores. While the C-NC model demonstrated the highest predictive value, the success rate of CTP scan in critically ill patients is often limited. Moreover, for AIS patients without large vessel occlusion, CTP may unnecessarily increase radiation exposure. In contrast, the C-NP model allows rapid assessment of the presence of large vessel occlusion and collateral status without requiring CTP. This model simplifies the preoperative workflow, achieves a higher examination success rate, offers broader applicability, and reduces radiation exposure for patients. Therefore, we propose that the C-NP model is the most suitable for preoperative assessment, providing clinicians with a practical tool to identify eligible candidates for MT.

### Limitations of this study include

(1) Stroke etiology data were not available in our study. Most patients came from rural or county-level areas with poor pre-admission healthcare access, and they were often unaware of their baseline vascular status. As a result, etiological classification could not be reliably determined within the short preoperative window, and the high rate of missing data precluded its inclusion. The absence of etiology may introduce unmeasured confounding and limit the generalizability of our models to populations with known or different etiological profiles. Future studies incorporating etiological stratification are needed to validate and refine our findings, which would enhance the model’s accuracy.

(2) Procedural variables (e.g., number of passes, device type, time to reperfusion, anesthesia type) and post-procedural management were not recorded or adjusted for. Because our model is intended for preoperative prediction, these factors are inherently unavailable at the time of prediction. However, their absence may affect model transportability, and future studies should integrate preoperative-available proxies or develop dynamic models that incorporate intra-procedural updates.

(3) The study population was selected based on corresponding diagnostic and therapeutic guidelines (59) and patient preference, making it difficult to avoid selection bias.

(4) Furthermore, the relatively small sample size necessitates further validation with larger cohorts. The present model was developed from a single-center cohort with a limited sample size. Although internal validation via cross-validation was performed to assess its performance, the risk of overfitting remains a concern. Its prospective and multi-center external validation is imperative before any widespread implementation can be contemplated. Our next step will involve conducting external validation across multiple stroke centers of different levels.

(5) It should be acknowledged that some studies have indicated that mTICI 2C/3 may confer better clinical outcomes than mTICI 2B (60). And advances in endovascular materials may facilitate more complete reperfusion in the future. Further research is needed to elucidate the effect of varying reperfusion grades on clinical prognosis.

(6) While our study used the Menon multiphase collateral score to assess arterial collateral status, we did not incorporate dedicated venous outflow models for comparison with other metrics. Recent literature has demonstrated that combined assessment of cortical and medullary venous outflow can improve the predictive performance of multiparametric models in stroke patients (61–63). Given that multiphase CTA data were available, future studies should explore whether integrating venous outflow assessment could provide additional prognostic value beyond arterial collateral grading alone. This remains an important direction for future research.

(7) Different CTP processing platforms employ proprietary algorithms for deconvolution, arterial input function selection, and baseline calibration. Consequently, even when applying the same relative cerebral blood flow (rCBF) < 30% threshold to define the ischemic core, absolute infarct volume measurements can differ substantially across platforms. This limits the direct comparability of our volumetric data with studies that used other software. Future studies should consider cross-platform validation or the use of standardized phantoms to harmonize measurements.

## Conclusion

The combined models based on multimodal CT demonstrated a robust predictive ability for the prognosis of patients with acute anterior circulation occlusive stroke following MT. The C-NP model streamlines the examination process, enhances assessment speed, and reduces patient radiation exposure, thereby enabling clinicians to better assess prognosis after successful intervention and holding considerable promise for broader adoption in primary care settings.

## Data Availability

The raw data supporting the conclusions of this article will be made available by the authors, without undue reservation.
