# Supplementary material for: Preoperative multimodal CT for selection of acute anterior circulation occlusion stroke patients for mechanical thrombectomy
Source: Front Neurol. 2026 May 8;17:1818377. doi: 10.3389/fneur.2026.1818377 (PMC13194055; doi:10.3389/fneur.2026.1818377)
Supplement: Supplementary file 1 [file Data_Sheet_1.PDF]

Table S1: univariate analysis of Patients with failed recanalization(mTICI≤2a)

Variate	effective（n=1）(%)	ineffective（n=9）(%)	$X^2/t/Z$	*P*value
**Male**	1(100)	7(78)	0.278	＞0.999
**Age（y）**	87	67.1±8.9	2.128	0.066
**Hypertension**	1(100)	5(56)	0.741	＞0.999
**Diabetes**	0(0)	1(11)	0.123	＞0.999
**AF**	1(100)	4(44)	0.665	＞0.999
**PBG（mmol/L）**	5.8	6.1（5.61，9.53）	-0.174	0.862
**NIHSS**	11	15±4	-0.871	0.409
**Length of thrombus（mm）**	12	20.3（17，32.2）	-1.571	0.116
**ASPECTS**	8	7±3	0.448	0.666
**Collateral score**	3	3（2，3）	-0.189	0.850
**Infarct core volume (ml)**	1.02	3.8（0，14.7）	-0.180	0.857
**Hypoperfusion volume (ml)**	4.41	100.9±72.9	-1.255	0.245
**Ischemic penumbra volume (ml)**	3.39	79.7±56.8	-1.275	0.238

Table S2: univariate analysis of thrombectomy refusal

Variate	effective（n=3）(%)	ineffective（n=34）(%)	$X^2/t/Z$	*P*value
**Male**	1(33)	19(56)	0.564	0.584
**Age（y）**	58(58,75)	66.5(48.5,73.5)	-0.111	0.066
**Hypertension**	3(100)	26(76)	0.901	＞0.999
**Diabetes**	1(33)	7(21)	0.264	0.530
**AF**	1(33)	12(35)	0.005	＞0.999
**PBG（mmol/L）**	5.8	6.1（5.61，9.53）	-0.668	0.504
**NIHSS**	8±6	17±7	-2.219	0.033
**Length of thrombus（mm）**	4.8(4.8,6.6)	13.9（9.6，22.8）	-2.059	0.040
**ASPECTS**	6(6,8)	3(1,5)	-1.376	0.169
**Collateral score**	3(3,4)	1(1,3)	-1.562	0.118
**Infarct core volume (ml)**	0(0,6.8)	4.9（0，29.3）	-1.145	0.252
**Hypoperfusion volume (ml)**	136.3±73.4	186.6±96.8	-0.873	0.388
**Ischemic penumbra volume (ml)**	123.5±57.1	122.5±53.9	0.032	0.974
